# Dr. Fisher’s Handbook of the Diseases of Children

**Published:** 1896-04

**Authors:** Montague R. Leverson


### DR. FISHER'S HANDBOOK OF THE DISEASES OF CHILDREN.

EDITOR HOMŒOPATHIC PHYSICIAN.

DEAR SIR:—I do not purpose entering into any general discussion of Dr. Fisher's *Handbook of the Diseases of Children*, reviewed in your issue for February (page 106), but I do desire to protest most earnestly against the *unscientific* character of his chapter on vaccination, and the *gross untruthfulness* of his statements and pretended statistics. The vitality of a lie is astonishing! To expose the misstatements contained in the first paragraph of page 242 of Dr. Fisher's work would take up too much of your space; the falsehoods of the second paragraph are not more flagrant, but can be exposed at a less cost of my time, of your space, and of your readers' patience.

The falsehood started in the German Parliament, where a member, who was also a vaccinating official, afflicted with that verophobia which is a characteristic disease of the class, placed the small-pox mortality of the French army at 23,469 during the Franco-Prussian war, and that of the German army at about 3,400 to 3,600.

This did not please the Anglican and American verophobists, who by successive "attenuations" have reduced the German small-pox mortality to 261. They did not dare raise the alleged 23,469 of the French army. Why they did not do so will be made clear presently.

The lie was presented to the Royal (British) Commission of 1889, by Dr. Arthur F. Hopkirk, in the form of 23,469 for France (Q. 1543 report of the Commission, p. 14 b.) and of 316 for Germany (Q. 1496, Ib. p. 11 b.). This 316 has been further "attenuated" by American verophobists (Qy. by Dr. Charles E. Fisher, President of the American Institute of Homœopathy ?) to 261.

The falsehood of these figures is so palpable that they ought not to have deceived a child; 23,469 deaths from small-pox means fully 180,000 cases! This fact alone is so absurdly monstrous that there is absolutely no excuse for Dr. Fisher not having investigated further before adopting the falsehood. Had he done so he would have found that all the figures upon the subject were pure fabrications. The statistics being disputed, inquiry was made by the Royal British Commission (through the intermediary of the British Foreign Office) of the various authorities in France and Germany as to the mortality from small-pox in the French and German armies respectively, during the Franco-Prussian war; from both countries came the unqualified reply that the information sought was inaccessible, because NO RECORD HAD BEEN KEPT of the diseases to which the troops succumbed!

This sample of blind copying—or worse—on the part of Dr. Fisher condemns his entire work. A work by a man incapable of discriminating, accepting as true a statement *false upon its face,* rehashing as arguments for a superstitious rite "*statistics*" whose falsity had been crushingly exposed, a rite, too, opposed to the fundamental principles of Homœopathy, a work by such a man is unworthy of serious criticism.

Bad as all this is, "worse remains behind." Further "attenuation" of the figures being hardly possible, Dr. Fisher has introduced a new feature of falsehood which may be termed a *Fisherism*.

No previous verophobist, in reciting the fable, had ventured to say that the French army was *unvaccinated*. This embellishment was reserved for this (pretended) *homœopathic* falsifier. The Anglo-Saxon monosyllable of three letters, however unfashionable, is the only word which will rightly designate it. So far from the French army being UNvacinated, every recruit, without one single exception, on entering the French army is *forcibly vaccinated*.

If the figures were true what greater proof of the uselessness of vaccination could be adduced?

The mendacity of these vaccinating quacks is appalling. Dr. Fisher's work must be abandoned to contempt.

Respectfully,

MONTAGUE R. LEVERSON.

